# Continuities of Risk in the Era of the Mental Capacity Act

**DOI:** 10.1093/medlaw/fww036

**Published:** 2016-12-22

**Authors:** John Fanning

**Affiliations:** School of Law and Social Justice, University of Liverpool, Liverpool, UK

**Keywords:** Deprivation, Liberty, Safeguards, Authorisation, Autonomy, Reform

## Abstract

When compared with the Mental Capacity Act (MCA) 2005, the Mental Health Act (MHA) 1983 seems an outlier. It authorises compulsory treatment of mental disorders on the basis of P’s risks. English law, therefore, discriminates between mental and physical disorders. Following the UK’s ratification of the Convention on the Rights of Persons with Disabilities (CRPD), the MHA probably also violates international law. Against this backdrop, one might expect that decisions contingent on risk are confined to the MHA and have no relevance elsewhere. This article argues that the opposite is true: risk-based decision-making has colonised MCA processes and plays a key role in determining the nature of P’s interaction with health services. These ‘continuities’ of risk are most notable in the Deprivation of Liberty Safeguards (DOLS), where assessments of risk are implicitly significant for best interests and eligibility determinations. Using governmentality theory as an explanatory model, this article claims that the DOLS can be reconstructed as part of a wider legal apparatus for the regulation of the risks of harm associated with mental disorders. The article also argues that the Law Commission’s recent proposals to introduce a new ‘protective care’ scheme and expand the remit of the MHA hint at a ‘rehabilitation’ of risk as an integral component of mental health and capacity law. The article concludes that the concept’s stigmatising potential, lack of definition, and conflict with the CRPD cast doubt on its capacity to reconcile English mental health law with the era of autonomy, capacity, and non-discrimination.

## 1. Introduction

Risk is integral to the Mental Health Act (MHA) 1983. To deploy its compulsory powers,^1^ two registered medical practitioners must certify that the patient (P) (i) is suffering from a mental disorder^2^ of the requisite nature or degree, and (ii) poses a risk of harm to his health or safety or to other people.^3^ Risk, therefore, has a transformative effect on P’s interaction with mental health services, turning it into a relationship defined by a paternalistic imperative.

The centrality of risk under the MHA contrasts sharply with the Mental Capacity Act (MCA) 2005. Here, P’s capacity to make decisions is the primary concern.^4^ The MCA has a broader scope than the MHA in that it applies to all patients, irrespective of the nature of their disorders. If P is unable to make a decision for himself then he will be deemed to lack capacity and a decision may be taken in his ‘best interests’.^5^ As the House of Lords Select Committee on the 2005 Act recently stated, the MCA is a ‘visionary’ piece of legislation that puts patients at the heart of decision-making processes.^6^

While the MHA authorises the detention of a mentally disordered person on the basis of risk, no equivalent power exists for physical disorders. As Richardson has argued, there seems to be no justification for singling out mental disorders in this way.^7^ Many commentators have criticised the involuntary care and treatment of mental disorders as a crude historical anachronism alien to a modern liberal state.^8^ Furthermore, the United Nations Convention on the Rights of People with Disabilities (CRPD) casts doubt on the MHA’s compatibility with the UK’s international legal obligations.^9^ Ten years since the introduction of the MCA, the MHA looks increasingly like an outlier. Consequently, one might expect that the domain of risk is narrowly confined to the MHA and that it plays a marginal role in care and treatment decisions.

This article makes an original contribution to the literature by evaluating the ‘continuities of risk’ in mental health and capacity law. Specifically, it focuses on the way that the Deprivation of Liberty Safeguards (DOLS) regime has amplified the significance of risk in mental health decision-making. The article advances two core claims. First, the concept of risk continues to determine the nature of P’s interaction with mental health services, even outside the MHA, and is implicitly relevant to determinations of ‘best interests’. Secondly, the Law Commission’s recent proposals to reform the law in this area would have expanded the domain of risk by bringing ‘informal’ patients (that is, patients suffering from a mental disorder and lacking capacity) within the scope of the MHA.^10^ Far from a containment of risk’s significance, the post-MCA era has witnessed the concept’s colonisation of mental health decision-making processes beyond the MHA.

This article is divided into five sections. Section II frames the debate by defining ‘risk’ for the purposes of mental health law and locating it within a theoretical context. It argues that the Foucauldian ‘governmentality’ thesis provides a compelling explanatory model for the utilisation of risk in mental health law. ‘Risk’ is an instrument of social control which provides a legitimate basis for the deployment of the state’s disciplinary powers against its citizens. Section III explores the extent to which the concept of risk informs the mechanics of the DOLS. Using the notion of an ‘escalator of risk’, it reconstructs the DOLS framework as part of a broader legal apparatus to regulate the risks of harm associated with mental illnesses. Section IV analyses the Law Commission’s reform proposals and argues that they would run counter to the presumed direction of travel in English mental health and capacity law. It also reflects on the revised proposals which appeared in the Law Commission’s recent ‘Interim Statement’.^11^ Section V concludes that a ‘rehabilitation’ of risk may be taking place, raising questions about where the balance lies between autonomy and paternalism in the post-MCA era.

## II. FRAMING THE DEBATE

The MHA does not define ‘risk’.^12^ Its admission criteria instead make oblique reference to the concept through their ‘risk formula’; that is, the words in the Act which render the deployment of the compulsory powers contingent on decision-makers’ evaluations of risk. There is nothing new about variations of the formula appeared in the original 1983 Act and its forerunners.^13^ The objective of avoiding or minimising the risks of harm associated with mental disorders has indisputably been at the core of English mental health law for some time.

It would be an oversimplification to argue that the MHA is concerned solely with the risk of violence. It is true that the scientific literature has explored the putative relationship between mental illness and violence in great depth.^14^ It is also true that this apparent relationship led to a renewed emphasis on risk as a policy driver during 1990s and 2000s.^15^ Yet the MHA’s risk formulae cover more than the risk of violence alone. First, the MHA Code of Practice contains factors which decision-makers should consider when deciding whether a person should be detained under the MHA.^16^ These factors include P’s risk of suicide, self-harm, self-neglect, deterioration, and jeopardising his health or safety accidentally, recklessly, or unintentionally.^17^ The Code, therefore, plainly acknowledges that the risk of violence is just one of a range of risks that can justify P’s compulsory admission to hospital.

Secondly, the MHA distinguishes between ‘risk’ and ‘dangerousness’. According to Lady Hale, the MHA does not require that P pose a *danger* as a prerequisite for admission to hospital.^18^ Dangerousness is a distinct concept relevant for the purposes of restricting the power of P’s nearest relative to discharge him.^19^ Lady Hale says that the absence of ‘danger’ from the MHA’s admission criteria indicates that they ‘were meant to be broader than those for keeping him there against the wishes of his family’.^20^ ‘Dangerousness’ implies a higher threshold than ‘risk’ under the MHA.^21^ Others have made similar distinctions. Pilgrim and Rogers, for example, argue that the MHA’s references to a patient’s ‘health or safety’ go further than danger and thereby legitimise the deployment of the ‘wide-ranging powers of [mental health] professionals’.^22^ Prins draws another (albeit less convincing) distinction between ‘risk’, which is the likelihood of an event occurring, and ‘danger’, which is the degree of damage that may result from it.^23^ In any case, ‘risk’ is not necessarily a synonym for ‘dangerousness’. ‘Danger’ suggests a heightened sense of urgency and tends to imply dangerousness to others. ‘Risk’, by contrast, goes much further, encompassing ‘reflexive’ hazards; that is, hazards that coalesce around or within the patient and whose effects will be limited thereto. This unambiguously incorporates more common hazards such as self-harm, self-neglect, abuse, exploitation, and so on, into the purview of the MHA, rendering them as legitimate occasions on which to justify compulsory interventions. By interpreting ‘risk’ in this way, the MHA’s underlying policy is about more than public protection. Considerations of risk and positive health outcomes are, therefore, much more closely linked than conventional wisdom might suggest.

While various risks of harm will legitimise a mentally disordered person’s compulsory admission to hospital, *why* the MHA should permit this paternalistic exception to the principle of self-determination is not obvious. Indeed, the MHA’s risk centricity discriminates between physical and mental disorders: why should doctors be able to treat a person with schizoaffective disorder on a compulsory basis and yet be prevented from doing the same to a person with, say, diabetes? It is submitted that the answer lies in the theoretical constructions of risk which inform Michel Foucault’s ‘governmentality’ thesis. ‘Governmentality’ describes a situation ‘in which the state becomes increasingly concerned with the government of a population as an end in itself rather than the consolidation of state power’.^24^ According to Foucault, the modern state exhibits a continuing interest in overseeing ‘the welfare of [its] population, the improvement of its condition, the increase of its wealth, longevity [and] health’.^25^ The state’s deployment of what Foucault called ‘biopower’^26^ began when the discovery of statistical regularities among populations led to ‘a fundamentally quantitative feel for nature, how it is and how it ought to be’.^27^ These statistics revealed that populations have their own regularities, their own rates of death and disease, cycles of scarcity, and so on.^28^ Foucault argued that states seek to integrate citizens ‘into systems of efficient and economic controls’ by supervising the population’s regularities and disciplining those that deviate from them.^29^ He believed in particular that mental illness was reconstructed as a ‘social danger’, which justified the deployment of coercive power as a method of ‘medical discipline’ directed at ‘transforming the individual’.^30^ For Foucault, the maintenance of a productive population is, above all else, the ‘ultimate end of government’.^31^ Coercive mental health laws are a means to that end.

Governmentality’s ‘constructivism’ assumes that risks are defined by ‘discourses’ through which ‘dominant institutions formulate language and information that generates and fuels prevalent ideas’.^32^ Lupton argues that imputations of risk are invariably levelled against people on the margins of society.^33^ She says that modern society values the ‘civilised body’ (ie that which is white, able-bodied, bourgeois, heterosexual, and masculine) over ‘The Other’ (eg women, the working class, non-whites, the disabled, and gays and lesbians (and, presumably, the mentally ill)).^34^ ‘The Other’ poses a risk and is, therefore, deemed to be ‘needful of control, surveillance and discipline’.^35^ This socially constructed interpretation contrasts with the realism of ‘risk society’ theory, which defines risks as the objectively identifiable, manufactured hazards of modernity.^36^ Yet the constructivist interpretation is crucial in the mental health context, where the risks which justify compulsory care and treatment may defy objective explication. This may go some way to explaining the ‘risk exceptionalism’ of the MHA. Even if the risks flow from other factors (as opposed to diagnosis alone), it is clear that psychiatric interventions are contingent on a calculus of risk.^37^ According to Castel, these interventions no longer even necessarily require a person to manifest symptoms of abnormality: ‘it is enough to display whatever characteristics the specialists … have constituted as risk factors’.^38^

‘Governmentality’ provides a theoretical context for enduring paternalistic imperatives in medico-legal discourse. The theory also explains why the concept of risk is fundamental to the mechanics of the MHA’s civil commitment powers. The rationale for the deployment of coercive power is to reduce or extinguish the risks which constitute deviations from the norm. Risk is, therefore, the occasion on which compulsory interventions are made under the MHA. Governmentality also explains why English law discriminates between physical and mental disorders by authorising involuntary treatment on the basis of risk for the latter but not the former. The risks of harm associated with mental disorders are constructed in such a way that society has customarily deemed them to be more deserving of coercive discipline than those associated with physical disorders. While this distinction is empirically flawed, Foucault’s theory illuminates the MHA’s unique place in the corpus of English medical law.

The contrast between the MHA and MCA is striking. The MCA generally does not authorise any person to deprive any other person of his liberty,^39^ emphasises the importance of an individual’s involvement in decisions about his welfare,^40^ and allows the relevant professionals to take decisions *for and on behalf of* the patient where appropriate. After 10 years, it might be reasonable to expect that the 2005 Act has delimited the domain of risk to the MHA. This article claims that the opposite is true: risk’s domain is not limited to the MHA proper; in fact, the concept has colonised decision-making processes outside the scope of the compulsory powers. These continuities of risk are most evident in the context of the DOLS. Parliament introduced the DOLS to plug the ‘*Bournewood* gap’^41^—the legal ‘no man’s land’^42^ between ‘formal’ patients (that is, those subject to the MHA) and ‘voluntary’ patients. Following *HL v United Kingdom*,^43^ any public hospital or care home in UK which held patients in *Bournewood-*style circumstances was responsible for multiple and continuing violations of Article 5 of the ECHR. To address this, policy-makers designed a conceptually distinct framework of legal safeguards for people deprived of their liberty in hospital but not subject to the MHA—the DOLS.^44^

It is submitted that the DOLS have amplified the significance of risk in mental health and capacity law. When viewed through the governmentality prism, the DOLS are part of a continuum of legal apparatus concerned with the assessment and management of the risks associated with mental disorders. Denney argues that a diagnosis of mental illness places a person on a figurative ‘escalator of dangerousness’, up and down which he will move at different moments in his life.^45^ This article adopts Denney’s ‘escalator’ as a useful way of locating the DOLS on the continuum of legal responses to risk. While the MHA plainly applies in higher risk situations, it is arguable that the DOLS cater for situations of risk which fall lower down the figurative escalator.

## III. DOLS AND CONTINUITIES OF RISK

Various commentators have condemned the DOLS as ‘hideous’,^46^ ‘obscure’,^47^ ‘overcomplicated’,^48^ ‘bureaucratic’, and at odds with the ‘elegant simplicity’ of the MCA.^49^ Yet the interrelationship between the DOLS and the MHA reveals much about the implicit significance of risk to the decision-making process. First, the MHA and the DOLS are mutually exclusive: P cannot be subject to the 1983 Act’s compulsory powers and the safeguards at the same time.^50^ Secondly, the fact that the DOLS are an exception to the general rule that the MCA cannot authorise deprivations of liberty suggests that they were designed to apply where there may be a heightened risk of harm to P.^51^ While these risks may not warrant recourse to the MHA, they are such that voluntary arrangements alone would be inadequate. Indeed, if ‘deprivation of liberty’ involves ‘complete supervision and control’ and the absence of the freedom to leave,^52^ it follows that the safeguards will apply in circumstances where mere restrictions on P’s freedom of movement may not be enough to attenuate the risks associated with her mental disorder.

### A*. DOLS, Risk,* *and Best Interests*

If a hospital wishes to deprive P of his liberty, it must apply for a ‘standard authorisation’^53^ in accordance with Schedule A1 to the MCA.^54^ This process must take place where P is (i) about to be or is already accommodated in a hospital or care home, (ii) likely to be a detained resident within the next 28 days, and (iii) likely to meet all of the six qualifying requirements^55^ in Part 3 of Schedule A1 to the MCA; namely age,^56^ mental health,^57^ mental capacity,^58^ best interests,^59^ eligibility,^60^ and no refusals.^61^

Some of the criteria are easier to assess than others. P must be at least 18 years of age to satisfy the age requirement,^62^ and the ‘no refusals’ criterion precludes an authorisation where P has refused some or all of the proposed treatment in an applicable advance decision^63^ or where his admission will conflict with a valid decision of a donee of a lasting power of attorney or a deputy appointed by the court.^64^ The mental health and mental capacity requirements are similarly straightforward: P must be suffering from a mental disorder within the meaning of section 1(2) of the MHA^65^ and must lack capacity to decide whether he should be accommodated in the relevant hospital or care home.^66^ Things get trickier when it comes to the best interests requirement. Here, the assessor must be satisfied that it is (i) in P’s best interests for him to be deprived of his liberty, (ii) necessary for P to be detained in order to prevent harm to him, and (iii) a proportionate response to the likelihood of P suffering harm and the seriousness of that harm.^67^ The wording here bears a striking similarity to the MHA’s risk formula. The DOLS provisions, therefore, require at least *some* degree of risk of harm before a standard authorisation can be granted. This is presumably not the same level of risk as that which is implied by the MHA. First, Schedule A1 to the MCA refers only to detention which is necessary to prevent harm *to the relevant person*. This is clearly a narrower and perhaps less urgent conception of risk than that which applies under the MHA, which impels decision-makers to take risks to the wider community into account too. Secondly, the DOLS provisions seem specifically to incorporate considerations of risk *into* the assessment of P’s best interests. To some extent, this is unsurprising: the MCA provides that where P lacks capacity the decision-maker should consider, inter alia, any factors that would likely influence his decision in order to give effect to his best interests.^68^ The decision-maker should, therefore, take a decision that is broadly commensurate with what P might decide in the same circumstances. The safeguards thus draw an inextricable link between reducing risks of harm to P and enhancing his best interests.^69^ No such link exists in the MHA. Through these two differences we can see how the DOLS framework further reinforces the niche for the MHA’s compulsory powers, which apply (i) where P poses or faces graver risks of harm, and (ii) according to a paternalistic imperative.

### B*. DOLS, Risk,* *and Eligibility*

The DOLS’ eligibility requirement further amplifies the significance of risk. According to paragraph 17(1) of Part 3 of Schedule A1 to the MCA, ‘the relevant person meets the eligibility requirement *unless* he is ineligible to be deprived of his liberty …’^70^ The simplest way in which P will be rendered ineligible is where, as we have seen, he is (i) subject to a hospital treatment regime, and (ii) detained in a hospital under that regime.^71^ The eligibility question becomes more complex where P is (i) within the scope of the MHA, but (ii) not subject to any of its provisions at the material time.^72^ P will be ‘within the scope’ of the MHA if (i) an application could be made in respect of him under section 2 or 3 of the 1983 Act, and (ii) he could be detained in hospital pursuant to such an application were one made.^73^ In these circumstances, P will be ineligible under the DOLS framework where (i) the standard authorisation would authorise him to be a mental health patient, (ii) P objects either to being a mental health patient or to being given some or all of the relevant treatment, and (iii) a donee or deputy has not made a valid decision to consent to each matter to which P objects.^74^ This means that a patient who is within the scope of the MHA but does not object to his admission to hospital or to an aspect of his treatment therein can notionally be the subject of the DOLS *or* the compulsory powers. It may be that the chosen regime is simply a matter of preference for the decision-maker.^75^ The presence of some form of objection, however, tips the scales in favour of the MHA.^76^

Here again it may be that considerations of risk determine which side of the line P falls. Where P objects, the risks of harm presumably exceed the level at which he can comfortably remain ‘informal’. Charles J confirmed this dynamic in *J v The Foundation Trust*,^77^ although His Lordship overstated the MHA’s superiority somewhat. Charles J said that the original purpose of the DOLS was to ‘leave the existing regime under the 1983 Act in place with primacy and to fill a gap left by it and the common law’.^78^ In *DN v Northumberland Tyne and Wear NHS Foundation Trust*,^79^ Jacobs J said that it is not possible to say in the abstract which of the DOLS and the MHA has priority over the other ‘without reference to the circumstances of the particular case’.^80^ While the MHA will have primacy over the DOLS where it applies, Jacobs J said that it is not true that the former will *always* take precedence. In this case, DN suffered from Alcohol Dependency Syndrome and was detained under section 3 of the MHA. He argued that he should instead be subject to the DOLS because it was not necessary for the purposes of section 3 to detain him for treatment. DN’s carers were using diversion and distraction techniques to prevent him drinking alcohol. Jacobs J held that this was not ‘specialist mental health care’ within the meaning of section 145(1) of the MHA and, therefore, it was neither appropriate nor necessary to detain DN under section 3. On the facts of DN’s case, the MHA did not have primacy over the MCA. Charles J reached a similar conclusion in *AM v South London and Maudsley NHS Foundation Trust*,^81^ where he revisited the ‘primacy’ principle he had set out in *J v Foundation Trust*. Here, His Lordship said that general propositions concerning the relationship between the MHA and the MCA are ‘dangerous’.^82^ Charles J acknowledged that the MHA’s primacy only arises as a matter of course where P is within the scope of the MHA but not subject to any of its provisions at the material time.^83^

If the choice between the DOLS and the MHA is a matter of fact then the *risks inherent in the circumstances* may determine which regime will apply. In *NM v Kent County Council*,^84^ the patient had a mild learning disability, behavioural difficulties, and a sexual interest in children. The dispute here arose over whether the First-tier Tribunal’s decision to continue NM’s guardianship arrangement under the MHA had been correct in law. Jacobs J held that the tribunal had acted within the jurisdiction conferred upon it by section 72(4) of the MHA. Significantly, His Lordship explained that the tribunal’s analysis justified the need for guardianship in NM’s case despite the notional availability of the DOLS as an alternative.^85^ Without the strictures of a guardianship order, NM would not remain in the home in which he was resident. This would have a detrimental effect on the continuity of his treatment which would, in turn, *increase the risks of harm* to children and to NM. The decision to continue NM’s guardianship arrangements was, therefore, justified *by the risks posed to and by him*. NM was higher up the figurative escalator of risk and, therefore, warranted the closer degree of oversight and supervision conferred by the MHA. A similar dynamic is discernible in *Hillingdon LBC v Neary,*^86^ albeit obliquely and at the lower end of the escalator. Here, a local authority deprived Steven, a 21-year-old man with severe autism, of his liberty in a residential support unit, despite evidence which suggested that it was in his best interests to remain at home. The court held that the local authority’s assessment of Steven’s best interests was flawed: it had not taken into account Steven’s wish to go home or his father’s request that he return to his care.^87^ Although the court did not express it in this way, it is difficult to escape the conclusion that the risks in Steven’s case did not warrant close supervision and control.

## IV. REFORMING DOLS, REHABILITATING RISK?

The House of Lords Select Committee, which carried out post-legislative scrutiny of the MCA, concluded in 2014 that the DOLS were ‘not fit for purpose’.^88^ The Law Commission has recently declared that there is a ‘compelling case’ for replacing the DOLS.^89^ Yet, as Bartlett has observed, talk of DOLS reform raises an important question: ‘What is it exactly that we want?’^90^ There is very little agreement about this. While the prevailing view may be that Parliament must reform the DOLS, there has up to now been no consensus about what a ‘post-DOLS’ framework might look like. One option is to focus first on what the safeguards are intended to achieve and then to design new provisions accordingly.^91^ Another solution is to bring informal patients within the ambit of the MHA’s guardianship provisions.^92^ Szerletics and O’Shea propose the introduction of a new First-tier Tribunal for Mental Capacity as a means of fulfilling the core functions of the DOLS.^93^ A less onerous reform would involve redrafting the MHA Code of Practice to clarify the mechanics of the DOLS.^94^

In 2015, the Law Commission proposed a ‘protective care’ scheme.^95^ The essence of this scheme was that it would adapt to the locus of P’s care arrangements, thereby departing from the ‘one-size-fits-all’ rigidity of the DOLS.^96^ It would comprise three mechanisms—namely ‘supportive care’, ‘restrictive care and treatment’, and a separate hospital-based scheme—and allow decision-makers to tailor their patients’ care arrangements according to their needs. In the first instance, all patients who lack capacity and live in care homes, supported living and shared lives accommodation would be subject to ‘supportive care’.^97^ The principal function of this ‘protective outer layer’ would be to reinforce existing support mechanisms without creating new legal machinery.^98^ Next, if P required more intrusive care or treatment then she would be subject to ‘restrictive care and treatment’.^99^ This component of the Law Commission’s proposals would have directly replaced the DOLS.^100^ If P’s carers proposed any form of restrictive care and treatment then the relevant local authority would initiate an assessment to determine that she lacks capacity to consent to it and that the treatment is in her best interests. Crucially, this assessment should also establish that the relevant restrictions represent the least restrictive option and that they are ‘necessary to prevent harm’.^101^ If the ‘Approved Mental Capacity Professional’^102^ were to decide that the basis for restrictive care and treatment has been met then she would be able to approve the arrangements for up to twelve months at a time.^103^

Where hospital-based care and treatment is concerned, the Law Commission proposed an entirely separate scheme.^104^ In contrast to the arrangements which it proposed for care homes, supported living and so on, this hospital scheme would be organised around the concept of deprivation of liberty.^105^ The justification for this distinct arrangement is that decision-making in hospitals is fundamentally different from that which occurs in the social care setting.^106^ Decisions in the social care context are ‘often made by teams in advance and over a period of time’; whereas in hospitals ‘decisions need to be made immediately, sometimes by a single clinician’.^107^ Furthermore, the Law Commission contended that the implications of hospital-based decisions can be different from those taken in the social care setting. For example, deprivations of liberty in care homes ‘may have permanent implications’, whereas those which take place in hospital settings are ‘more likely to be of shorter duration and may have less irreversible effects’.^108^

Where patients lacking capacity and suffering from mental disorders are concerned, the Law Commission’s provisionally preferred solution was ‘to construct a solution based in the Mental Health Act’.^109^ In other words, the Law Commission proposed to extend the MHA specifically to incorporate ‘informal’ patients.^110^ It contended that this would ‘hopefully establish a clear-cut interface’ between the MCA and the MHA, and ‘remove the issues [surrounding] objection and treatment and the purpose of … admission’.^111^

The Law Commission’s original proposals were striking for two reasons. First, they appeared to anticipate that the intrusiveness and intensity of the ‘protective care’ scheme would depend on assessments of risk, among other things. The Law Commission stated that deciding whether the ‘relevant restrictions are necessary to prevent harm’ should be part of an assessment of a patient’s best interests.^112^ It also left open the possibility that an evaluation of the risks of harm a person may pose to others should become a core component of the ‘best interests’ assessment.^113^ Where a person lacks capacity and the relevant risks of harm are low, the scheme would subject her to basic ‘supportive care’ arrangements. Where the risks may be greater, an Approved Mental Capacity Professional might opt for a ‘restrictive care and treatment’ arrangement. This might include continuous or complete supervision and control; significant restrictions over the person’s diet, clothing, or access to and contact with his relatives, carers, and friends; or restrictions on the patient’s freedom of movement.^114^ It is difficult to see how considerations of risk would not figure in determining the level of supervision and control that might be required. The ‘escalator of risk’ analogy, therefore, seems quite apposite.

Secondly, the proposal to amend the 1983 Act so that it would provide a legal mechanism for depriving ‘informal’ patients of their liberty in hospital would bring an entirely new constituency of patients within the scope of the MHA. In order to differentiate between the civil commitment powers and the proposed hospital-based scheme, an amended MHA would presumably have to incorporate a lower risk threshold. For example, an amended MHA might incorporate welfare considerations where informal patients are concerned, thereby echoing the guardianship provisions^115^ and expressly legitimising deprivations of liberty intended to tackle or prevent the risks of self-neglect, abuse, exploitation, self-harm, and so on. This expansion in the domain of risk would surely retrench the principles of autonomy and non-discrimination, effectively reversing the trends which have informed medico-legal debates in recent years.

The Law Commission’s proposals for a ‘protective care’ scheme would have done little to curtail the continuities of risk which characterise the current legal framework. Indeed, ‘protective care’ would have expanded the domain of risk. In this way, the scheme chimed with the governmentality theory, in that it ultimately relied on similar conceptual foundations to the MHA to effect a form of ‘discipline’. In its recent ‘Interim Statement’, published following an extensive consultation exercise involving hundreds of stakeholders, the Law Commission has revised its proposals. First, it has decided to narrow the focus of any new statutory scheme ‘on ensuring that those deprived of their liberty have appropriate and proportionate safeguards’.^116^ The scheme would, therefore, not go as far as the ‘protective care’ regime the Law Commission originally put forward. Secondly, the Law Commission appears to have rowed back from its original proposal to insert a new mechanism into the MHA which would cater for the admission to hospital of compliant, incapacitated patients.^117^ Yet the ‘Interim Statement’ still appears to anticipate that assessments of risk will play a determinative role; for example, it expects that the relevant decision-makers should give proper consideration to ‘the necessity of removing individuals from their own home and placing them in institutional care in the name of their best interests’.^118^ This raises an important question: should risk continue to function as a core component of the legal framework?

### A*. The Pragmatic Case for Risk*

The concept of risk could play an important role in allocating patients with mental disorders to suitable care and treatment arrangements. Informal patients are already typically at a lower risk of harm than formal patients. According to the Care Quality Commission (CQC), the highest number of applications for standard authorisations in 2014–15 came from care homes (80%), whereas the lowest came from specialist mental health hospitals (10%).^119^ Recent data from the Health and Social Care Information Centre (HSCIC) show that dementia accounted for more than half of all DOLS authorisations in UK in 2014–15.^120^ Older people are, therefore, much more likely than younger people to be subject to the DOLS, with the rate of applications for people aged 85 years and over nearly doubling since 2009.^121^ According to Clare’s research, mental health professionals tend to distinguish between ‘active’ medical treatment, which they see as a matter for the MHA, and ‘care’, which is one for the DOLS.^122^ It is obvious why this dichotomy exists: DOLS patients tend to have conditions which require long-term care, constitute a lower risk of harm, and which affect their cognitive functions. A typical DOLS patient is likely, therefore, to be older, resident in a care or nursing home, and suffering from dementia. She is also more likely to be female.^123^ These characteristics are not readily associated with high risks warranting compulsory admission to hospital under the MHA. By contrast, the archetypal MHA patient tends to be younger and is more likely to be male. He is by definition more likely than a DOLS patient to suffer from an acute psychiatric illness, such as depression or schizophrenia. Data from the HSCIC show that around two-thirds of patients detained under the MHA on 31 March 2014 were male^124^ and that fully 56% of all compulsory detentions in 2013–14 affected patients aged between 25 and 54 years.^125^ The typical MHA patient is more likely to display the sort of characteristics which are associated with higher levels of risk.^126^ According to guidance issued by the Department of Health in 2007, for example, young men with mental disorders were considered more likely to perpetrate violence than those with different demographic characteristics or clinical histories.^127^ Similar configurations of factors may also prompt patients to commit suicide or self-harm.^128^

This is not to say that there are *no* risks for decision-makers to take into account when assessing informal patients. It seems, however, that these risks are much less urgent and are, therefore, unworthy of detention and forcible treatment. A care scheme which escalates according to considerations of risk (among other things) would allow decision-makers to tailor the clinical responses to their patient’s needs. To a certain extent, of course, the interface between the MHA and the DOLS already achieves this; we have already seen the way risk implicitly acts as an organising principle in the decision-making process. Yet it does so imperfectly, creating a degree of overlap between the two regimes which causes unnecessary confusion and complexity. The Law Commission’s proposals would make it much easier for practitioners to navigate the legal landscape.

The second argument in favour of a ‘protective care’ scheme is that it would effectively abolish the confusing interface between the MHA and the DOLS while still ensuring compliance with Article 5 ECHR. As we have seen, whether or not a patient ‘objects’ is key. Yet ‘objection’ has proved to be a problematic concept. According to the MHA Code of Practice, whether a patient is objecting must be considered ‘in the round, taking into account all the circumstances, so far as they are reasonably ascertainable’.^129^ This raises more questions than it answers: How will decision-makers determine whether a patient, who lacks capacity, is objecting? Should they interpret ‘objection’ narrowly so that it applies only to plainly articulated refusals? If so, what about patients whose refusals may be fleeting or unpredictable?^130^ What about patients who lack the faculties of speech or movement?^131^ Alternatively, should decision-makers view anything less than passive acquiescence as an objection? If so, would there be any meaningful distinction between the absence of capacity and objection?^132^ As Richardson has written, ‘objection’ without further definition does not provide ‘an adequate basis on which to allocate vulnerable individuals to very different legal regimes’.^133^ Perhaps to cater for this, the Code of Practice states that if there is any doubt then the relevant decision-makers must conclude that P is objecting.^134^ Brindle and Branton suggest that this bias in favour of the MHA may already exist among mental health practitioners.^135^ No doubt many practitioners are drawn to the more familiar provisions of the MHA for pragmatic reasons.^136^

Escalating protective interventions according to assessments of risk may make the interface between the MHA and the MCA easier to navigate. Consider the *Bournewood* case as an example. Allen questions whether L would even have been eligible for a standard authorisation under the DOLS had they existed at the material time.^137^ He suggests that having regard to all of the reasonably ascertainable circumstances in the *Bournewood* case, it may be difficult to conclude that L was doing anything other than manifesting his objections. Yet, the risks that L may have posed or which he may have faced were apparently not deemed to be sufficient to justify his compulsory admission under the MHA: he did not resist his admission to the hospital, made no attempt to leave, and was compliant. A framework of safeguards designed around risk would have accommodated L much more readily than perhaps even the DOLS would have done. By determining how to care and treat P using an escalator of risk, doctors would no longer have to work out whether P objects to his care and treatment arrangement.

### B*. The Problem with Risk*

A framework that replaces the DOLS by expanding the reach of the MHA or which tolerates continuities of risk will inevitably be problematic. There are three reasons for this. First, the concept of risk is inherently stigmatising. Because considerations of risk are the principal trigger to compulsory admission to hospital under the MHA, there is an enduring link between mental illnesses and adverse outcomes. An expansion of the operational domain of the MHA would therefore do very little to de-stigmatise mental illness, a point which the Government has recognised in its preliminary response to the Law Commission’s consultation paper.^138^ In fact, such an expansion may serve to stigmatise a larger number of people, including those ‘informal’ patients who have hitherto fallen outside the scope of the 1983 Act. Any attempt to ‘rehabilitate’ risk as a structurally significant consideration in mental health law must confront this reality. It is doubtful whether it is even possible to divest the concept of its stigmatising connotations while simultaneously utilising it as a central organising component of mental health law. Consequently, the Law Commission’s decision not to proceed with an expansion of the MHA’s remit should be welcomed.

Secondly, the absence of a definition of ‘risk’ means that the concept is afflicted by a crippling lack of certainty. How we define risk or, more accurately, how we frame the risks with which we are concerned, will surely have a bearing on how the law operates. If we interpret ‘risk’ to mean ‘the chance that P will exhibit violence towards others or self-harm’ then the deployment of the compulsory powers will be contingent solely on the grave risks that the *patient* poses. Yet, as we have seen, considerations of risk can also relate to the chance that a person with a mental disorder might be unable to care for himself, suffer abuse, face exploitation, and so on. If ‘risk’ is framed in this way then the deployment of the compulsory powers might also, or alternatively, depend on the risks which others pose *to the patient*. Furthermore, the chance that the patient’s mental health might deteriorate, that his medication might cause him to suffer side-effects, that his therapeutic relationship with his clinical team might collapse, and so on, could all easily be articulated using the language of risk. Without defining ‘risk’, or at least specifying which risks have primacy, the MHA and other risk-based frameworks create the potential for ‘conflicts of risk’. For example, imagine that P has a mental disorder which may warrant admission for treatment under section 3 of the MHA. His clinical team believes that were P to be admitted to hospital on a compulsory basis there is a risk that his mental health will deteriorate further. It also recognises that P’s mental disorder means that he poses a risk of harm to other people. How should P’s clinical team resolve this conflict of risk? There is nothing in the MHA to suggest that some risks are more worthy of compulsory intervention than others and P’s doctors could easily make the case for ‘sectioning’ him *or* maintaining the status quo.^139^ Building a legal framework around the concept of risk without defining its content in any meaningful way leaves its operational priorities open to question.^140^

It is unsurprising that in the context of mental health law references to ‘risk’ are instinctively associated with violence. If ‘risk’ is mainly interpreted as ‘risk of harm to others’ then any escalation in the intensity of clinical interventions must necessarily correlate to an increased likelihood that a patient will exhibit violent behaviour. Yet ‘risk’ could just as easily relate to P’s ‘vulnerability’; that is, her mental disorder and incapacity might increase the risk that a third party will cause harm to her. It is, therefore, possible to describe a vulnerable patient as being ‘at risk’; indeed, the more vulnerable she is, the greater the risk becomes, and the stronger the case is for more restrictive interventions or deprivations of liberty. Yet this does not necessarily follow: it is unlikely that the most vulnerable patients require the most coercive interventions. What this demonstrates is that defining ‘risk’ is crucial if it is to play any meaningful role in organising the law’s mechanics. If ‘risk’ is associated with harm to others then a legal framework characterised by escalating restrictiveness is a vehicle for public protection; if it is associated with ‘vulnerability’ then it fails to map onto patients’ care needs. Whether it is possible to select a definition of ‘risk’ that would provide a satisfactory and coherent basis on which to organise mental health and capacity law seems doubtful.

Finally, the continuing significance of risk conflicts with the UK’s obligations under the CRPD. The Convention requires States Parties to recognise that ‘all persons are equal before and under the law and are entitled without any discrimination to the equal protection and equal benefit of the law’.^141^ As Bartlett has written, it is difficult to see how UK mental health legislation as it stands can be remotely compliant with the CRPD’s principle of non-discrimination.^142^ Having ratified the Convention in 2009, the UK is obliged ‘to modify or abolish existing laws, regulations, customs and practices that constitute discrimination against persons with disabilities’.^143^ One might, therefore, expect that the upshot of this obligation would be the abolition of the MHA and a radical reimagining of the nature and purpose of mental health and capacity law. The Law Commission’s original proposals for a ‘protective care’ scheme and an expansion of the MHA pulled in the opposite direction. In the post-CRPD era, proposals such as these seem intrinsically problematic: how can legal frameworks whose very operation turns on considerations of a mentally disordered person’s incapacity or risk possibly function in accordance with the principle of non-discrimination?

They probably cannot. Although it is true that Article 11 of the CRPD requires States Parties to take ‘all necessary measures to ensure the protection and safety of persons with disabilities in situations of risk’, this seems to preclude compulsory interventions on the basis of the sort of risks that are currently relevant under the MHA. The continuities of risk which exist at the interface of the MHA and DOLS are, therefore, fundamentally at odds with the CRPD. Even the Law Commission’s revised proposals to reform the DOLS would do very little to correct this. For example, Article 12(2) requires States Parties to recognise that persons with disabilities ‘enjoy legal capacity on an equal basis with others in all aspects of life’. The operation of any post-DOLS scheme seems to depend on (in)capacity being an occasion on which to deploy the law’s protective function. Such a scheme would conflict with the UK’s general obligation under Article 4(1)(e) of the CRPD ‘to eliminate discrimination on the basis of disability …’. According to Article 14(1)(b), States Parties must also ensure that the existence of a disability shall in no case justify a deprivation of liberty. The Law Commission’s proposals would seem directly to contradict this provision.

The Law Commission’s ‘protective care’ scheme assumed that the intensity of the law’s intervention would depend on considerations of risk among other things. Its proposal to amend the MHA to apply to ‘informal’ patients would have significantly expanded the domain of risk beyond its original limits. Far from abandoning considerations of risk in favour of a non-discriminatory approach to mental health care and treatment, the proposals seem to conflict with recent trends. Consequently, ‘rehabilitating’ risk is profoundly problematic: it poses significant policy, practical and legal challenges which bring the very legal basis of any framework organised around the concept into question.

## V. CONCLUSIONS

In the 10 years since the MCA was introduced, the MHA has become an outlier. Its policy objective to tackle the risks of harm associated with mental disorders clashes with the principles which underpin the MCA. Its discriminatory effects are likely to violate the UK’s obligations under the CRPD. One might, therefore, expect that decisions beyond the MHA are no longer solely contingent on risk of harm and that concept’s domain has been narrowly delimited. This article has shown that in fact the opposite is true. Considerations of the risk of harm continue to determine the nature and extent of P’s interaction with mental health services, even outside the context of the MHA.

These continuities of risk are most evident in relation to the DOLS. There, perceived risks of harm are impliedly relevant to determining P’s best interests and his eligibility to be deprived of his liberty. ‘Governmentality’ theory explains why the concept of risk has colonised processes beyond the MHA in this way. It posits that states enact health laws to serve a disciplinary function with respect to risk. That the DOLS exist on a continuum with the MHA makes it possible to reconstruct them as part of a wider legal apparatus for managing the risks of harm associated with mental disorders. That the DOLS-MHA interface operates according to an ‘escalator’ dynamic further reinforces this impression; the bigger the risks, the more coercive the intervention.

Perhaps more surprising is the content of the Law Commission’s recent proposals to reform mental health and capacity law. First, the ‘protective care’ scheme as it was originally conceived would also have escalated according to considerations of P’s risks of harm. Secondly, the proposal to expand the MHA to incorporate ‘informal’ Ps within its domain would have constituted a significant extension of the scope of compulsory mental health law. Even the Law Commission’s revised proposals appear to anticipate that considerations of risk should play a determinative role in any post-DOLS statutory regime. Far from retrenching risk-based processes in favour of non-discriminatory alternatives, the Law Commission’s proposals would propagate the continuities of risk.

Any such ‘rehabilitation’ of risk is likely to be controversial. On one hand, considerations of risk allow decision-makers to tailor their interventions to P’s clinical need, thereby personalising his care and treatment. On the other hand, risk poses significant challenges in the post-MCA era. It is stigmatising, poorly defined, and probably breaches the CRPD. More must be done to reconcile English mental health law with the principles of capacity, autonomy, and non-discrimination. It is doubtful that the concept of risk could ever be compatible with or instrumental to that reconciliation.

## Footnotes

1 These include the detention of P in hospital for a relevant period (ss 2–5; 20), a requirement that P lives in a particular place under the supervision of a guardian (s 8(1)(a)), the administering of medical treatment to P without his consent (s 63), and so on.

2 MHA 1983, s 1(2): ‘mental disorder’ means ‘any disorder or disability of the mind’.

3 The wording of the ‘risk formula’ varies according to which of the compulsory powers the registered medical practitioners wish to deploy. For example, s 2 says that the application must be justified on the grounds that the patient ‘ought to be detained in the interests of his own health or safety or with a view to the protection of other persons’, whereas s 3 says detention must be ‘necessary for the health or safety of the patient or for the protection of other persons’.

4 MCA 2005, s 2(1): a person lacks capacity if, at the material time, ‘he is unable to make a decision for himself … because of an impairment of, or a disturbance in the functioning of, the mind or brain’.

5 MCA 2005, ss 3 and 4. Case law predating the 2005 Act suggests that the court will evaluate P’s best interests broadly; see eg *Re MB (Medical Treatment)* [1997] 2 FLR 426, in which the Court of Appeal said that considerations of patients’ best interests should not be limited only to clinical matters; *Re A (Male Sterilisation)* [2000] 1 FLR 549, in which Butler-Sloss LJ said that ‘best interests’ include ‘medical, emotional and all other welfare issues’; *Trust A v H* [2006] 9 CCLR 474, in which ‘best interests’ was taken to refer to a ‘broad spectrum of medical, social, emotional and welfare issues’.

6 House of Lords Select Committee on the Mental Capacity Act 2005, *Mental Capacity Act 2005: Post-legislative Scrutiny*, Report of Session 2013–14 (TSO, HL Paper 139, 2014) 6.

7 G Richardson, ‘Balancing Autonomy and Risk: A Failure of Nerve in England and Wales’ (2007) 30 Intl JL Psychiat 71, 73. Cf AE Buchanan and DW Brock, *Deciding for Others: The Ethics of Surrogate Decision-making* (CUP 1990) 328–29.

8 S Rosenman, ‘Mental Health Law: An Idea Whose Time has Passed’ (1994) 28 Aust NZ J Psychiat 560, 565; G Szmukler and F Holloway, ‘Reform of the Mental Health Act: Health or Safety?’ (2000) 177 Brit J Psychiat 196, 198; T Campbell and C Heginbotham, *Mental Illness: Prejudice, Discrimination and the Law* (Dartmouth 1991) 7.

9 P Bartlett, ‘The United Nations Convention on the Rights of Persons with Disabilities and Mental Health Law’ (2012) 75(5) MLR 752; F Morrissey, ‘The United Nations Convention on the Rights of Persons with Disabilities: A New Approach to Decision-making in Mental Health Law’ (2012) 19(5) EJHL 423; G Szmukler and others, ‘Mental Health Law and the UN Convention on the Rights of Persons with Disabilities’ (2014) 37(3) Intl JL Psychiat 245.

10 Law Commission, *Mental Capacity and Deprivation of Liberty: A Consultation Paper* (TSO, Consultation Paper No 222, 2015).

11 Law Commission, *Mental Capacity and Deprivation of Liberty: Interim Statement*, 25 May 2016 <www.lawcom.gov.uk/wp-content/uploads/2016/06/mental_capacity_interim_statement.pdf> accessed 22 June 2016.

12 ‘Risk’ appears on only a handful of occasions: ss 17A(1)(6), 17B(2)(b), 17E(1)(b), 20A(7), 41(1), 43(1)(b), and 72(1A).

13 Mental Health Act 1959, ss 25(1)(b), 26(2)(b), 43(3), 123(1) and (2); Lunacy Act 1890, s 4.

14 See eg J Monahan, ‘Mental Disorder and Violent Behaviour’ (1992) 47(4) Am Psychol 511; MG Kennedy, ‘Relationship Between Psychiatric Diagnosis and Patient Aggression’ (1993) 14(3) Issues Ment Health Nurs 263; BG Link and A Stueve, ‘Psychotic Symptoms and the Violent/Illegal Behaviour of Mental Patients Compared to Community Controls’ in J Monahan and others (eds), *Violence and Mental Disorder: Developments in Risk Assessment* (University of Chicago Press 1994); E Silver and others, ‘Assessing Violence Risk Among Discharged Psychiatric Patients: Towards an Ecological Approach’ (1999) 23(2) Law & Hum Behav 237; J Swanson and others, ‘Violent Behaviour Preceding Hospitalisation Among Persons with Severe Mental Illness’ (1999) 23(2) Law & Hum Behav 185; RD Hare, ‘Psychopathy and Risk for Recidivism and Violence’ in N Gray and others (eds), *Criminal Justice, Mental Health and the Politics of Risk* (Cavendish Publishing 2002); JW Swanson and others, ‘The Social-Environmental Context of Violent Behaviour in Persons Treated for Severe Mental Illness’ (2002) 92(9) Am J Publ Health 1523; JW Swanson and others, ‘A National Study of Violent Behaviour in Persons with Schizophrenia’ (2006) 63 Arch Gen Psychiat 490.

15 N Glover-Thomas, ‘The Age of Risk: Risk Perception and Determination Following the Mental Health Act 2007’ (2011) 19 Med L Rev 581, 585; see also HM Government, *Reforming the Mental Health Act – Part I: The New Legal Framework* (Cm 5016-I, December 2000); HM Government, *Reforming the Mental Health Act – Part II: High Risk Patients* (Cm 5016-II, December 2000); JM Laing, ‘Rights versus Risk? Reform of the Mental Health Act 1983’ (2000) 8 Med L Rev 210; R Daw, ‘The Mental Health Act 2007: The Defeat of an Ideal’ (2007) 16 JMHL 131; N Glover-Thomas, ‘The Mental Health Act 2007 in England and Wales: The Impact on Perceived Patient Risk Profiles’ (2010) 29(4) JL & Med 593.

16 Department of Health, *Mental Health Act 1983: Code of Practice* (TSO 2015) [14.10–14.11].

17 ibid.

18 B Hale, *Mental Health Law* (5th edn, Sweet and Maxwell 2010) 58.

19 MHA 1983, s 25(1).

20 Hale (n 18).

21 See also *R (on the application of O) v West London Mental Health NHS Trust* [2005] EWHC 604 (Admin), where Collins J said that the term ‘dangerous’ in s 25(1) requires decision-makers to look for an ‘extra factor’ before barring a nearest relative’s discharge order.

22 D Pilgrim and A Rogers, ‘Two Notions of Risk in Mental Health Debates’ in T Heller and others (eds), *Mental Health Matters: A Reader* (Palgrave MacMillan 1996) 183.

23 H Prins, ‘Risk Assessment and Management in Criminal Justice and Psychiatry’ (1996) 7(1) J Forensic Psychiatr 42, 44.

24 D Denney, *Risk and Society* (SAGE 2005) 35.

25 M Foucault, ‘Governmentality’ in G Burchell and others (eds), *The Foucault Effect: Studies in Governmentality* (University of Chicago Press 1991) 100.

26 M Foucault, *The Will to Knowledge: The History of Sexuality – Volume 1* (Penguin Books 1998) 136, 139.

27 I Hacking, *The Taming of Chance* (CUP 1990) 5; see also I Hacking, ‘How Should we do the History of Statistics?’ in G Burchell and others (eds), *The Foucault Effect: Studies in Governmentality’* (University of Chicago Press 1991).

28 Foucault (n 25) 99.

29 Foucault (n 26).

30 M Foucault, ‘About the Concept of the ‘Dangerous Individual’ in 19^th^ Century Legal Psychiatry’ (1978) 1 Intl JL Psychiat 1, 7, 10.

31 ibid.

32 G Mythen, ‘Sociology and the Art of Risk’ (2008) 2(1) Sociol Compass 299, 306.

33 D Lupton, *Risk* (Routledge 1999) 3, 49.

34 ibid 130.

35 ibid 147.

36 See eg U Beck, *Risk Society: Towards A New Modernity* (SAGE 1992); A Giddens, *The Consequences of Modernity* (Polity 1990); Mythen (n 32); U Beck, *World Risk Society* (Polity 1999); U Beck, *World At Risk* (Polity 2009); F Warner and others, *Risk: Analysis, Perception and Management* (Royal Society 1992); U Beck, ‘Politics of Risk Society’ in J Franklin (ed), *The Politics of Risk Society* (Polity 1998); P Strydom, *Risk Environment and Society: Ongoing Debates, Current Issues, and Future Prospects* (Open UP 2002).

37 H Kemshall, *Risk, Social Policy and Welfare* (OUP 2002) 102; Denney (n 24) 115–16.

38 R Castel, ‘From Dangerousness to Risk’ in G Burchell and others (eds), *The Foucault Effect: Studies in Governmentality* (University of Chicago Press 1991) 288.

39 MCA 2005, s 4A(1).

40 MCA 2005, s 1.

41 *R v Bournewood Community and Mental Health NHS Trust, ex parte L* [1998] UKHL 24.

42 P Bartlett, ‘Informal Admissions and Deprivation of Liberty under the Mental Capacity Act 2005’ in L Gostin and others (eds), *Principles of Mental Health Law and Policy* (OUP 2010) 386.

43 (2005) 40 EHRR 32.

44 Department of Health, Bournewood *Briefing Sheet*, Gateway Reference: 6794, June 2006, 2.

45 Denney (n 24) 115–16.

46 Bartlett (n 42) 392.

47 R Hargreaves, ‘The Deprivation of Liberty Safeguards – Essential Protection or Bureaucratic Monster?’ (2009) 19 JMHL 117, 124.

48 L Series, ‘Deprivation of Liberty Safeguards: a Haphazard Affair’ *The Guardian* (London, 2 April 2012) <http://www.guardian.co.uk/social-care-network/2012/apr/02/deprivation-liberty-safeguards-improvements> accessed 28 January 2013.

49 House of Lords Select Committee on the Mental Capacity Act 2005 (n 6) 91.

50 Ministry of Justice, *Mental Capacity Act 2005 – Deprivation of Liberty Safeguards: Code of Practice to Supplement the Main Mental Capacity Act 2005 Code of Practice* (TSO 2008) [1.11].

51 MCA 2005, ss 4A(2)(a) and 4A(5).

52 *Cheshire West and Cheshire Council v P* [2014] UKSC 19, [54] (Baroness Hale).

53 See pt 4 of sch A1 to the MCA 2005.

54 para 2 of pt 1 of sch A1.

55 para 12 of pt 3 of sch A1.

56 ibid, para 12(1)(a).

57 ibid, para 12(1)(b).

58 ibid, para 12(1)(c).

59 ibid, para 12(1)(d).

60 ibid, para 12(1)(e).

61 ibid, para 12(1)(f).

62 para 13 of pt 3 and para 34 of pt 4 of sch A1.

63 para 19(1) and (2) of pt 3 and para 48 of pt 4 of sch A1.

64 para 20(1)–(3) of pt 3 and para 48 of pt 4 of sch A1.

65 Importantly, the exclusion of learning disabilities from the MHA’s definition of ‘mental disorder’ under s 1(2A) of the 1983 Act does not apply to the DOLS. para 14(1) of pt 4 and para 35 of pt 4 of sch A1.

66 para 15 of pt 3 and para 37 of pt 4 of sch A1.

67 para 16(2)–(5) of pt 3 and paras 38 and 39 of pt 4 of sch A1; see also MCA 2005, s 4.

68 MCA 2005, s 4(6)(a)–(c).

69 For further discussion on this relationship, see Buchanan and Brock (n 7); MR Wicclair, ‘Patient Decision-making Capacity and Risk’ (1991) 5(2) Bioethics 91; I Wilks, ‘The Debate over Risk-related Standards of Competence’ (1997) 11(5) Bioethics 413.

70 Emphasis added.

71 ‘Case A’ in the table under para 2 of pt 1 of sch 1A to the MCA 2005.

72 ‘Case E’ in the table under para 2 of pt 1 of sch 1A.

73 para 12(1)(a) and (b) of pt 2 of sch 1A.

74 para 5(2)–(5) of pt 2 of sch 1A.

75 P Bartlett, ‘Civil Commitment’ in L Gostin and others (eds), *Principles of Mental Health Law and Policy* (OUP 2010) 471.

76 para 6 of pt 2 of sch 1A.

77 [2009] EWHC 2972 (Fam).

78 ibid [60].

79 [2011] UKUT 327 (AAC).

80 ibid [20].

81 [2013] UKUT 0365 (AAC).

82 ibid [78].

83 See n 72.

84 [2015] UKUT 125 (AAC).

85 ibid [24].

86 [2011] EWHC 1377 (COP).

87 ibid [182] (Peter Jackson J).

88 House of Lords Select Committee on the Mental Capacity Act 2005 (n 6) [257].

89 Law Commission (n 10) [2.41].

90 P Bartlett, ‘Reforming the Deprivation of Liberty Safeguards (DOLS): What Is It Exactly that We Want’ (2014) 20(3) Web JCLI 1. <http://webjcli.org/article/view/355/465> accessed 15 February 2015.

91 ibid.

92 R Robinson and L Scott-Moncrieff, ‘Making Sense of *Bournewood*’ (2005) 12 JMHL 17; R Jones, ‘Deprivation of Liberty: Mental Health Act or Mental Capacity Act’ (2007) 16 JMHL 170.

93 A Szerletics and T O’Shea, *The Deprivation of Liberty Safeguards: Essex Autonomy Project Briefing* (Essex Autonomy Project 2011) 30.

94 HM Government, *Valuing Every Voice, Respecting Every Right: Making the Case for the Mental Capacity Act* (Cm 8884, June 2014) [7.24].

95 Law Commission (n 10) ch 3.

96 ibid [2.23].

97 ibid [5.3], ch 6.

98 ibid.

99 ibid [5.3], ch 7.

100 ibid [7.1].

101 ibid [7.29].

102 This new designation would replace the current ‘best interests assessor’. ibid [7.71].

103 ibid [7.76].

104 ibid, chs 8 and 10.

105 ibid [8.20].

106 ibid [8.5].

107 ibid.

108 ibid [8.6].

109 ibid [10.21].

110 ibid [10.27].

111 ibid [10.21].

112 ibid [7.29].

113 ibid [7.42].

114 ibid [7.31].

115 MHA 1983, s 7(1)(b) provides that a guardianship application may be made in respect of a patient on the grounds that ‘it is necessary in the interests of the welfare of the patient or for the protection of other persons that the patient should be [received into guardianship]’.

116 Law Commission (n 11) [1.37].

117 ibid [1.45].

118 ibid [1.41].

119 CQC, *Monitoring the Use of the Mental Capacity Act Deprivation of Liberty Safeguards in 2014/15*, 30 <http://www.cqc.org.uk/sites/default/files/20151209_deprivation_of_liberty_safeguards_2014-15.pdf> accessed 25 April 2016.

120 HSCIC, *Deprivation of Liberty Safeguards (DoLS) Annex B: Granted Applications Data Tables* (HSCIC 2015) table 4.

121 ibid.

122 ICH Clare and others, *Understanding the Interface between the Mental Capacity Act’s Deprivation of Liberty Safeguards and the Mental Health Act* (University of Cambridge 2013) [3.5.7].

123 Law Commission (n 11), table 3.

124 HSCIC, *Patients Formally Detained in Hospitals under the Mental Health Act 1983 and Patients Subject to Supervised Community Treatment*, Annual Report, England, 2013–14, 14.

125 ibid 22.

126 See n 14; see also VA Hiday, ‘The Social Context of Mental Illness and Violence’ (1995) 36(2) J Health Soc Behav 122; N Rose, ‘Governing Risky Individuals: The Role of Psychiatry in New Regimes of Control’ (1999) 5(2) Psychiat Psychol & L 177.

127 Department of Health, *Best Practice in Managing Risk: Principles and Evidence for Best Practice in the Assessment and Management of Risk to Self and Others in Mental Health Services*, June 2007, app 2.

128 ibid.

129 Department of Health (n 16) [13.51].

130 M McKillop and others*,* ‘The Concept of Objection under the DOLS Regime’ (2011) 21 JMHL 61, 61.

131 ibid 68.

132 GS Owen and others*,* ‘Mental Capacity and Psychiatric In-patients: Implications for the New Mental Health Law in England and Wales’ (2009) 195 Brit J Psychiat 257, 261.

133 G Richardson, ‘Mental Capacity at the Margin: The Interface between Two Acts’ (2010) 18(1) Med L Rev 56, 73.

134 ibid.

135 N Brindle and T Branton, ‘Interface between the Mental Health Act and Mental Capacity Act: Deprivation of Liberty Safeguards’ (2010) 16 Adv Psychiatr Treat 430, 436; cf A Shah and C Heginbotham, ‘Newly Introduced Deprivation of Liberty Safeguards: Anomalies and Concerns’ (2010) 34(6) Psychiatr 243.

136 DP Herlihy and F Holloway, ‘The Mental Health Act and the Mental Capacity Act: Untangling the Relationship’ (2008) 8(12) Psychiatry 478, 480.

137 N Allen, ‘The *Bournewood* Gap (as Amended?): *GJ v Foundation Trust, Primary Care Trust, Secretary of State for Health’* (2010) 18(1) Med L Rev 78, 84.

138 HM Government, *Department of Health Response to the Law Commission’s Consultation on Mental Capacity and Deprivation of Liberty*, 11 December 2015 <https://www.gov.uk/government/publications/deprivation-of-liberty-standards-dols-consultation-response/department-of-health-response-to-the-law-commissions-consultation-on-mental-capacity-and-deprivation-of-liberty> accessed 7 April 2016.

139 Although see *W v Egdell* [1990] Ch 359 (CA), which suggests that where there is such a conflict of risk, the public’s interest in safety will outweigh the patient’s interests.

140 It is noteworthy that an expert committee appointed by the Department of Health in 1998 suggested that any new mental health law would ‘need to define [its] key concepts’ and ‘indicate the nature of risk assessment required’. Department of Health, *Report of the Expert Committee: Review of the Mental Health Act 1983*, November 1999, [5.103].

141 CRPD, art 5(1).

142 Bartlett (n 9) 754.

143 CRPD, art 4(1)(b). art 1 of the CRPD says ‘persons with disabilities’ include those who have long-term physical, mental, intellectual, or sensory impairments.

